# Monoclinic polymorph of chlorido­(dimethyl sulfoxide-κ*O*)tri­phenyl­tin(IV)

**DOI:** 10.1107/S2056989018000439

**Published:** 2018-01-12

**Authors:** Serigne Fallou Pouye, Ibrahima Cissé, Libasse Diop, Francisco Javier Ríos-Merino, Sylvain Bernès

**Affiliations:** a Laboratoire de Chimie Minérale et Analytique, Département de Chimie, Faculté des Sciences et Techniques, Université Cheikh Anta Diop, Dakar, Senegal; b Centro de Química, Instituto de Ciencias, Benemérita Universidad Autónoma de Puebla, 72570 Puebla, Pue., Mexico; c Instituto de Física, Benemérita Universidad Autónoma de Puebla, Av. San Claudio y 18 Sur, 72570 Puebla, Pue., Mexico

**Keywords:** crystal structure, polymorphism, tin, dimethyl sulfoxide, conformation

## Abstract

The crystal structure of the title tin complex, [Sn(C_6_H_5_)_3_Cl(C_2_H_6_OS)], (I), has been reported with one mol­ecule in the asymmetric unit in an ortho­rhom­bic cell [Kumar *et al.* (2009 ▸). *Acta Cryst.* E**65**, m1602–m1603]. While using SnPh_3_Cl as a starting material for a reaction for which the products were recrystallized over a very long time (six months) from dimethyl sulfoxide (DMSO), a new polymorph was obtained for (I), with two independent mol­ecules in the asymmetric unit of a monoclinic cell. The coordination geometry of the Sn centres remains unchanged, with the Cl^−^ ion and the DMSO mol­ecule in the apical positions and the phenyl C atoms in the equatorial positions of a trigonal bipyramid. The main difference between the polymorphs is the relative orientation of the phenyl rings in the equatorial plane, reflecting a degree of free rotation of these groups about their Sn—C bonds. In the crystal, mol­ecules are linked into [010] chains mediated by weak C—H⋯O inter­actions.

## Abstract 2

A new polymorph of [Sn(C_6_H_5_)_3_Cl(C_2_H_6_OS)] has been characterized, which crystallizes in space group *P*2_1_ with *Z*′ = 2, while the previously reported phase was in space group *P*2_1_2_1_2_1_ with *Z*′ = 1.

## Chemical context   

The Dakar research group and others worldwide have been focusing for a long time on the study of inter­actions of ammonium salts of oxyacids with metallic halides, to obtain adducts and complexes in which the oxyanion behaves as a ligand through its O atoms (Diassé-Sarr & Diop, 2011 ▸; Pouye *et al.*, 2014 ▸; Toure *et al.*, 2016 ▸; Sarr *et al.*, 2016 ▸; Ng & Hook, 1999 ▸). The main advantage of this general strategy is the high solubility of the ammonium salts in common organic solvents, which facilitates the development of traditional synthetic methods in solution. The well-known flip side is that separation and purification procedures are almost always necessary, and that such syntheses are not in line with the principles of Green Chemistry, since solvent is an intrinsic waste.

However, from time to time, when the recrystallization is the method of purification, as-yet undiscovered polymorphs of unreacted materials, products or by-products, are emerging. In such instances, the involved chemistry may be of little inter­est, while the chemical crystallography of the unexpected polymorph(s) may be of significant inter­est, even in borderline cases like the *disappearing polymorphs* (Bučar *et al.*, 2015 ▸). Actually, the propensity of a given mol­ecule to crystallize in various polymorphic forms is still difficult to predict (Price, 2009 ▸), and, for example, Ostwald’s ‘law of stages’ that states it is the least stable polymorph that crystallizes first, is of limited inter­est for concrete crystallizations (Threlfall, 2003 ▸). The current situation is thus that a significant number of new polymorphs are still obtained serendipitously, using a technique that could be coined as *crystallization by oblivion*. The herein reported title compound, (I), a new monoclinic polymorph of a frequently used starting material in tin chemistry, was obtained in this way: in one of our research programs, we have initiated the study of the inter­actions between [CH_3_NH_2_(CH_2_)_2_NH_2_CH_3_]SO_4_ and SnPh_3_Cl in a mixture of CH_2_Cl_2_ and dimethyl sulfoxide (DMSO) as solvent. One of the products obtained in an attempt of crystallization carried out over a very long time was the adduct obtained by addition of DMSO to the starting material SnPh_3_Cl, to form [SnPh_3_Cl(DMSO)]. The crystal structure of this compound has been reported previously, in space group *P*2_1_2_1_2_1_ (Kumar *et al.*, 2009 ▸; CSD refcode: RUGYOI, Groom *et al.*, 2016 ▸). In that case, crystals were obtained by dissolving SnPh_3_Cl in hot DMSO, affording fine colourless crystals by solvent evaporation over three days.
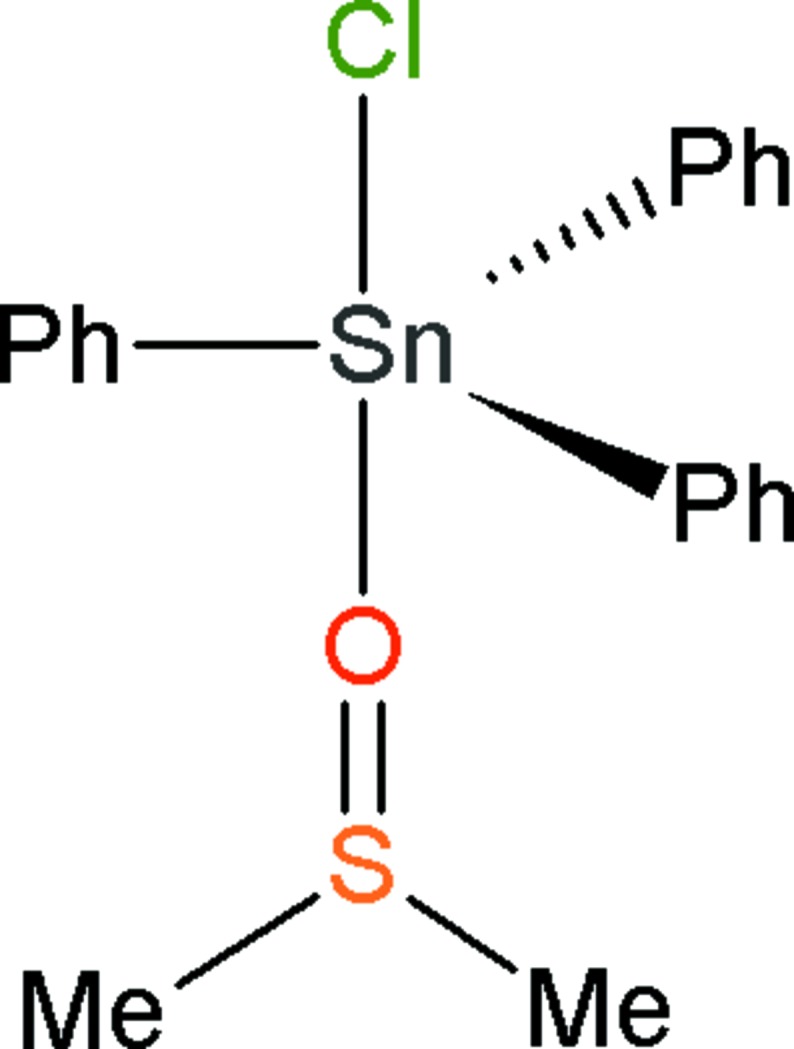


## Structural commentary   

**Figure 1:**
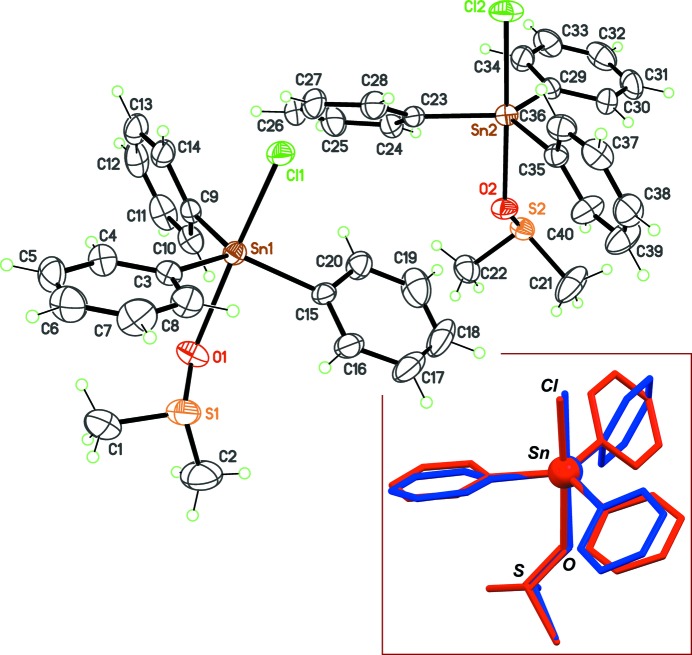
The asymmetric unit for the new monoclinic phase of the title compound, with displacement ellipsoids at the 30% probability level. The inset is a fit between independent mol­ecules, based on all non-H atoms (Macrae *et al.*, 2008 ▸), evidencing the rotation of one phenyl ring.

Instead of the known ortho­rhom­bic structure of the title compound, we crystallized a monoclinic polymorph, in space group *P*2_1_, with two mol­ecules in the asymmetric unit (Fig. 1 ▸).

**Figure 2:**
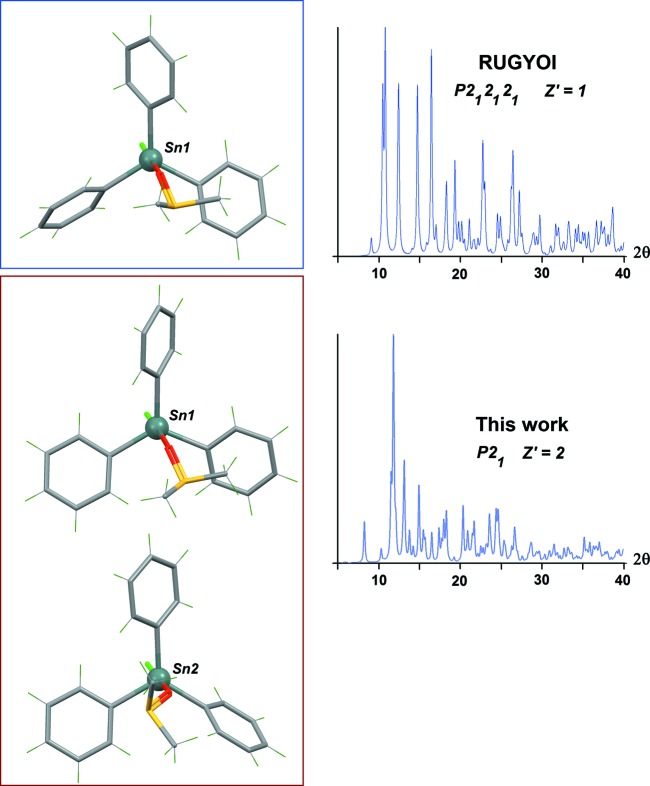
A comparison of the observed conformers for the title compound, viewed down the Cl—Sn—O axis (top: the previously known polymorph; bottom: the new *P*2_1_ polymorph). Note the different orientations observed for the apical DMSO mol­ecule. The calculated powder patterns displayed on the right show that both polymorphs are crystallographically very different. Patterns were calculated with *Mercury* (Macrae *et al.*, 2008 ▸; 5 < 2θ < 40°, λ = 1.54056 Å, FWHM = 0.2°).

The independent mol­ecules display different conformations, as a consequence of a degree of free rotation of the phenyl groups about their Sn—C bonds. An overlay between both mol­ecules gives deviations as high as 1.7 Å, and the rotation of one phenyl group is obvious (Fig. 1 ▸, inset). This conformational flexibility seems to be the reason why the compound has at least two stable polymorphs, even if the trigonal–bipyramidal geometry for the Sn centre is retained. The relative orientation of the phenyl rings in the observed conformers may be estimated using the dihedral angles formed by the rings in each mol­ecule. These angles span a large range, from 28.3 (4) to 87.2° (Table 1 ▸). As a consequence, the orientation of the DMSO mol­ecule with respect to the SnPh_3_ core is also variable. In the ortho­rhom­bic phase, the S—Me groups of DMSO are staggered with the Sn—C bonds; in the new monoclinic phase, one complex displays a similar conformation, while in the other the S—Me groups are eclipsed with the Sn—C bonds (Fig. 2 ▸). The resulting simulated powder diffraction patterns for each polymorph are, as expected, also very different (Fig. 2 ▸).

With such contrasting features for the dimorphic phases of [SnPh_3_Cl(DMSO)], obtained basically from DMSO solutions using short and long evaporation times, one could expect the apparition of other phases under different conditions of crystallization, for example by varying the solvent or the temperature of crystallization.

## Supra­molecular features   

**Figure 3:**
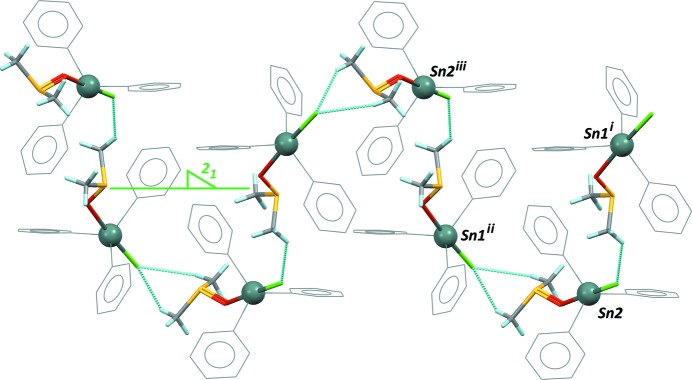
Part of the crystal structure of the title polymorph, showing the supra­molecular network formed along the screw axis 2_1_ in space group *P*2_1_. Dashed bonds represent C—H⋯Cl inter­molecular contacts. [Symmetry codes: (i) −1 + *x*, *y*, −1 + *z*; (ii) 1 − *x*, −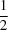 + *y*, 1 − z; (iii) −*x*, −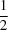 + *y*, −*z*.]

In the extended structure of the ortho­rhom­bic phase, one methyl group in DMSO forms weak C—H⋯Cl and C—H⋯π inter­actions, and mol­ecules related by the 2_1_ screw axis in the [010] direction feature π–π inter­actions between two phenyl rings, separated by 3.934 (3) Å (Kumar *et al.*, 2009 ▸). In the monoclinic form, mol­ecules related through the 2_1_ axis in space group *P*2_1_ no longer form π–π inter­actions. The supra­molecular structure of (I) is based rather on weak C—H⋯Cl contacts involving, as in the first polymorph, the methyl groups of the DMSO mol­ecule as donor, with H⋯Cl separations ranging from 2.82 to 2.94 Å. The resulting supra­molecular one-dimensional structure is a zigzag chain of alternating Sn1 and Sn2 independent mol­ecules, running along the screw axis (Fig. 3 ▸). The absence of other stabilizing inter­molecular contacts may suggest a less thermodynamically stable crystal, compared to the ortho­rhom­bic crystal obtained by fast crystallization, in contradiction with Ostwald’s rule (Threlfall, 2003 ▸). However, the crystal structures are in agreement with the calculated densities for both polymorphs: 1.562 g cm^−3^ for the ortho­rhom­bic form and 1.514 g cm^−3^ for the less stable monoclinic form reported here.

## Database survey   

According to the CSD (V5.39; Groom *et al.*, 2016 ▸), DMSO is a good coordinating solvent for tin: 64 hits may be recovered, in which the average value for the bond length Sn—O is 2.27 (11) Å for 105 instances. The bond length characterizing the coordination of DMSO in the monoclinic polymorph is very long compared to this average: the bond lengths Sn1—O1 and Sn2—O2 are 2.487 (4) and 2.368 (4) Å, respectively, reflecting a coordination of limited strength. Again, the ortho­rhom­bic form seems to be stabilized by comparison with the monoclinic form, as the DMSO is more tightly coordinated, with Sn—O(DMSO) = 2.311 (3) Å (Kumar *et al.*, 2009 ▸).

## Synthesis and crystallization   

[CH_3_NH_2_(CH_2_)_2_NH_2_CH_3_]SO_4_ has been synthesized on allowing CH_3_NH(CH_2_)_2_NHCH_3_ to react with H_2_SO_4_ in water in a 1:1 ratio. Slow evaporation of the resulting solution at 300 K gave after six weeks a yellowish viscous liquid supposed to be [CH_3_NH_2_(CH_2_)_2_NH_2_CH_3_]SO_4_ (**L**). When **L** (0.024 g, 0.130 mmol) dissolved in 50 ml of a 1:1 water/ethanol mixture was reacted with SnPh_3_Cl (0.100 g, 0.260 mmol) dissolved in a 1:1 di­chloro­methane/methanol mixture (50 ml), a slightly cloudy solution was obtained and filtered. The filtrate, when submitted to a slow solvent evaporation at 300 K over three days, produced a powder, which was redissolved in DMSO. Slow solvent evaporation at 300 K over six months afforded colourless blocks of (I) suitable for X-ray diffraction.

## Refinement   

Crystal data, data collection and structure refinement details are summarized in Table 2 ▸. The C-bound H atoms were included in calculated positions (C—H = 0.93–0.96 Å) and refined as riding, with *U*_iso_(H) =1.5*U*_eq_(C-methyl) and 1.2*U*_eq_(C) for other H atoms. The absolute configuration was assigned on the basis of the refinement of the Flack parameter (Parsons *et al.*, 2013 ▸).

## Supplementary Material

Crystal structure: contains datablock(s) I. DOI: 10.1107/S2056989018000439/hb4193sup1.cif

Structure factors: contains datablock(s) I. DOI: 10.1107/S2056989018000439/hb4193Isup2.hkl

CCDC reference: 1815199

Additional supporting information:  crystallographic information; 3D view; checkCIF report

## Figures and Tables

**Table 1:** Relative orientation (°) of the phenyl rings in the three conformers of the title mol­ecule Rings are arbitrarily labelled φ_*i*_ (*i* = 1, 2, 3) to compute the dihedral angles δ_i_. For (I), δ_i_ angles were calculated with *SHELXL2016/6* (Sheldrick, 2015*b* ▸).

Dihedral angle	*P*2_1_2_1_2_1_ phase^*a*^	*P*2_1_ phase, mol­ecule 1	*P*2_1_ phase, mol­ecule 2
δ_1_ = φ_1_/φ_2_	63.5	65.1 (2)	53.6 (3)
δ_2_ = φ_2_/φ_3_	70.7	65.1 (2)	59.1 (3)
δ_3_ = φ_1_/φ_3_	87.2	28.3 (4)	39.2 (3)

Note: (*a*) Kumar *et al.*, 2009 ▸.

**Table 2:** Experimental details

Crystal data	
Chemical formula	[Sn(C_6_H_5_)_3_Cl(C_2_H_6_OS)]
*M*_r_	463.57
Crystal system, space group	Monoclinic, *P*2_1_
Temperature (K)	297
*a*, *b*, *c* (Å)	8.81934 (18), 15.3698 (3), 15.4209 (3)
β (°)	103.294 (2)
*V* (Å^3^)	2034.31 (7)
*Z*	4
Radiation type	Mo *K*α
μ (mm^−1^)	1.49
Crystal size (mm)	0.48 × 0.30 × 0.23

Data collection	
Diffractometer	Rigaku OD Xcalibur Atlas Gemini
Absorption correction	Analytical (*CrysAlis PRO*; Rigaku OD, 2015 ▸)
*T*_min_, *T*_max_	0.880, 0.941
No. of measured, independent and observed [*I* > 2σ(*I*)] reflections	133515, 14767, 10835
*R*_int_	0.051
(sin θ/λ)_max_ (Å^−1^)	0.767

Refinement	
*R*[*F* ^2^ > 2σ(*F* ^2^)], *wR*(*F* ^2^), *S*	0.038, 0.083, 1.04
No. of reflections	14767
No. of parameters	437
No. of restraints	1
H-atom treatment	H-atom parameters constrained
Δρ_max_, Δρ_min_ (e Å^−3^)	1.48, −0.75
Absolute structure	Flack *x* determined using 4338 quotients [(*I* ^+^)−(*I* ^−^)]/[(*I* ^+^)+(*I* ^−^)] (Parsons *et al.*, 2013 ▸)
Absolute structure parameter	−0.039 (6)

Computer programs: *CrysAlis PRO* (Rigaku OD, 2015 ▸), *SHELXT2014* (Sheldrick, 2015*a* ▸), *SHELXL2016* (Sheldrick, 2015*b* ▸), *XP* in *SHELXTL* (Sheldrick, 2008 ▸) and *Mercury* (Macrae *et al.*, 2008 ▸).

## supplementary crystallographic information

### Crystal data

**Table:**

[Sn(C_6_H_5_)_3_Cl(C_2_H_6_OS)]	*F*(000) = 928
*M**_r_* = 463.57	*D*_x_ = 1.514 Mg m^−^^3^
Monoclinic, *P*2_1_	Mo *K*α radiation, λ = 0.71073 Å
*a* = 8.81934 (18) Å	Cell parameters from 28302 reflections
*b* = 15.3698 (3) Å	θ = 3.3–25.8°
*c* = 15.4209 (3) Å	µ = 1.49 mm^−^^1^
β = 103.294 (2)°	*T* = 297 K
*V* = 2034.31 (7) Å^3^	Block, colourless
*Z* = 4	0.48 × 0.30 × 0.23 mm

### Data collection

**Table:**

Rigaku OD Xcalibur Atlas Gemini diffractometer	14767 independent reflections
Radiation source: Enhance (Mo) X-ray Source	10835 reflections with *I* > 2σ(*I*)
Graphite monochromator	*R*_int_ = 0.051
Detector resolution: 10.5564 pixels mm^-1^	θ_max_ = 33.0°, θ_min_ = 3.0°
ω scans	*h* = −13→13
Absorption correction: analytical (CrysAlis PRO; Rigaku OD, 2015)	*k* = −23→23
*T*_min_ = 0.880, *T*_max_ = 0.941	*l* = −23→23
133515 measured reflections	

### Refinement

**Table:**

Refinement on *F*^2^	Secondary atom site location: difference Fourier map
Least-squares matrix: full	Hydrogen site location: inferred from neighbouring sites
*R*[*F*^2^ > 2σ(*F*^2^)] = 0.038	H-atom parameters constrained
*wR*(*F*^2^) = 0.083	*w* = 1/[σ^2^(*F*_o_^2^) + (0.0301*P*)^2^ + 1.1572*P*] where *P* = (*F*_o_^2^ + 2*F*_c_^2^)/3
*S* = 1.04	(Δ/σ)_max_ = 0.001
14767 reflections	Δρ_max_ = 1.48 e Å^−^^3^
437 parameters	Δρ_min_ = −0.74 e Å^−^^3^
1 restraint	Absolute structure: Flack *x* determined using 4338 quotients [(*I*^+^)-(*I*^-^)]/[(*I*^+^)+(*I*^-^)] (Parsons *et al.*, 2013)
0 constraints	Absolute structure parameter: −0.039 (6)
Primary atom site location: structure-invariant direct methods	

### Fractional atomic coordinates and isotropic or equivalent isotropic displacement parameters (Å^2^)

**Table:**

	*x*	*y*	*z*	*U*_iso_*/*U*_eq_	
Sn1	0.34055 (4)	0.62754 (2)	0.81374 (2)	0.04086 (8)	
Cl1	0.14377 (15)	0.71185 (10)	0.70653 (11)	0.0575 (3)	
S1	0.6727 (2)	0.60605 (13)	0.98769 (13)	0.0760 (5)	
O1	0.5508 (5)	0.5538 (3)	0.9246 (3)	0.0626 (10)	
C1	0.6394 (12)	0.5930 (10)	1.0910 (6)	0.154 (7)	
H1A	0.530448	0.600927	1.088428	0.232*	
H1B	0.698577	0.635231	1.130756	0.232*	
H1C	0.670287	0.535548	1.112350	0.232*	
C2	0.8465 (9)	0.5461 (9)	1.0021 (7)	0.121 (3)	
H2A	0.829588	0.488105	1.021127	0.182*	
H2B	0.926307	0.573706	1.046403	0.182*	
H2C	0.878378	0.543618	0.946699	0.182*	
C3	0.3347 (6)	0.7082 (3)	0.9248 (3)	0.0440 (11)	
C4	0.2591 (8)	0.6773 (4)	0.9877 (4)	0.0589 (15)	
H4	0.209888	0.623450	0.978833	0.071*	
C5	0.2546 (9)	0.7242 (5)	1.0634 (5)	0.0741 (19)	
H5	0.202098	0.702700	1.104703	0.089*	
C6	0.3290 (10)	0.8032 (6)	1.0764 (5)	0.084 (2)	
H6	0.329355	0.834620	1.127994	0.101*	
C7	0.4025 (9)	0.8362 (5)	1.0150 (6)	0.084 (2)	
H7	0.449645	0.890623	1.023832	0.101*	
C8	0.4066 (8)	0.7885 (4)	0.9395 (5)	0.0661 (16)	
H8	0.458269	0.810722	0.898147	0.079*	
C9	0.2087 (6)	0.5120 (3)	0.8110 (3)	0.0419 (11)	
C10	0.2788 (9)	0.4313 (4)	0.8150 (4)	0.0579 (14)	
H10	0.385218	0.426973	0.818618	0.070*	
C11	0.1885 (11)	0.3558 (4)	0.8136 (4)	0.075 (2)	
H11	0.236112	0.301569	0.816462	0.090*	
C12	0.0321 (11)	0.3608 (5)	0.8082 (5)	0.080 (2)	
H12	−0.026361	0.310414	0.808045	0.096*	
C13	−0.0371 (9)	0.4402 (6)	0.8030 (5)	0.076 (2)	
H13	−0.143832	0.443817	0.798711	0.091*	
C14	0.0488 (7)	0.5163 (4)	0.8039 (4)	0.0578 (14)	
H14	−0.000695	0.570040	0.799842	0.069*	
C15	0.5039 (5)	0.6303 (4)	0.7325 (3)	0.0449 (10)	
C16	0.6395 (7)	0.6785 (4)	0.7522 (5)	0.0625 (15)	
H16	0.665734	0.709282	0.805463	0.075*	
C17	0.7363 (8)	0.6816 (6)	0.6940 (7)	0.087 (2)	
H17	0.825710	0.715735	0.707348	0.105*	
C18	0.7026 (10)	0.6354 (7)	0.6177 (7)	0.099 (3)	
H18	0.769586	0.637220	0.579073	0.119*	
C19	0.5686 (12)	0.5852 (6)	0.5964 (6)	0.097 (3)	
H19	0.546093	0.552816	0.544026	0.116*	
C20	0.4687 (8)	0.5835 (4)	0.6535 (4)	0.0616 (15)	
H20	0.377499	0.550909	0.638875	0.074*	
Sn2	0.21662 (4)	0.54546 (2)	0.32336 (2)	0.04487 (8)	
Cl2	−0.03762 (16)	0.62413 (13)	0.27444 (12)	0.0729 (4)	
S2	0.52976 (17)	0.39472 (10)	0.33520 (9)	0.0526 (3)	
O2	0.4552 (4)	0.4702 (3)	0.3724 (3)	0.0534 (9)	
C21	0.7148 (10)	0.4347 (6)	0.3252 (8)	0.111 (4)	
H21A	0.700502	0.475938	0.277182	0.166*	
H21B	0.777566	0.387183	0.313190	0.166*	
H21C	0.766040	0.462650	0.379761	0.166*	
C22	0.5951 (9)	0.3236 (4)	0.4253 (5)	0.0731 (18)	
H22A	0.651875	0.355947	0.475644	0.110*	
H22B	0.661969	0.280355	0.408952	0.110*	
H22C	0.507284	0.295784	0.440433	0.110*	
C23	0.1457 (6)	0.4846 (4)	0.4317 (3)	0.0465 (11)	
C24	0.1469 (7)	0.3946 (4)	0.4382 (4)	0.0604 (14)	
H24	0.186122	0.361348	0.398009	0.073*	
C25	0.0900 (9)	0.3541 (6)	0.5046 (5)	0.082 (2)	
H25	0.087611	0.293635	0.507276	0.098*	
C26	0.0380 (9)	0.4017 (8)	0.5655 (5)	0.091 (3)	
H26	0.002901	0.374122	0.610813	0.109*	
C27	0.0370 (9)	0.4904 (7)	0.5604 (5)	0.084 (2)	
H27	0.000894	0.522937	0.602374	0.101*	
C28	0.0893 (7)	0.5321 (5)	0.4932 (4)	0.0683 (16)	
H28	0.086386	0.592521	0.489635	0.082*	
C29	0.1821 (6)	0.4685 (4)	0.2058 (3)	0.0449 (11)	
C30	0.2624 (8)	0.4840 (5)	0.1406 (4)	0.0627 (16)	
H30	0.333652	0.529506	0.147514	0.075*	
C31	0.2388 (10)	0.4330 (6)	0.0654 (5)	0.081 (2)	
H31	0.292424	0.445230	0.021473	0.097*	
C32	0.1357 (10)	0.3636 (6)	0.0544 (5)	0.083 (2)	
H32	0.122027	0.328401	0.004200	0.099*	
C33	0.0542 (9)	0.3475 (5)	0.1184 (5)	0.077 (2)	
H33	−0.016318	0.301658	0.111762	0.093*	
C34	0.0780 (7)	0.4004 (4)	0.1930 (4)	0.0608 (15)	
H34	0.021684	0.389495	0.236020	0.073*	
C35	0.3540 (6)	0.6620 (4)	0.3340 (4)	0.0477 (12)	
C36	0.2896 (8)	0.7420 (4)	0.3393 (4)	0.0596 (15)	
H36	0.184343	0.745718	0.339215	0.072*	
C37	0.3757 (9)	0.8177 (4)	0.3449 (5)	0.0664 (17)	
H37	0.329304	0.871349	0.349298	0.080*	
C38	0.5283 (9)	0.8128 (5)	0.3439 (5)	0.0745 (19)	
H38	0.586515	0.863655	0.347323	0.089*	
C39	0.5983 (9)	0.7348 (5)	0.3381 (6)	0.087 (2)	
H39	0.703561	0.732256	0.337942	0.104*	
C40	0.5110 (8)	0.6591 (5)	0.3323 (6)	0.074 (2)	
H40	0.557948	0.605751	0.327231	0.089*	

### Atomic displacement parameters (Å^2^)

**Table:**

	*U*^11^	*U*^22^	*U*^33^	*U*^12^	*U*^13^	*U*^23^
Sn1	0.04052 (16)	0.03857 (15)	0.04383 (16)	−0.00402 (14)	0.01042 (12)	−0.00110 (15)
Cl1	0.0418 (6)	0.0626 (8)	0.0669 (8)	0.0044 (6)	0.0100 (6)	0.0178 (7)
S1	0.0559 (9)	0.0893 (13)	0.0750 (11)	0.0037 (8)	−0.0011 (8)	−0.0046 (9)
O1	0.065 (2)	0.055 (2)	0.057 (2)	0.007 (2)	−0.0088 (18)	0.000 (2)
C1	0.082 (6)	0.30 (2)	0.070 (5)	−0.013 (8)	−0.003 (5)	−0.019 (8)
C2	0.066 (5)	0.174 (10)	0.117 (7)	0.033 (6)	0.008 (5)	−0.025 (8)
C3	0.046 (3)	0.040 (2)	0.044 (3)	0.001 (2)	0.006 (2)	−0.003 (2)
C4	0.072 (4)	0.048 (3)	0.059 (4)	0.004 (3)	0.019 (3)	−0.002 (3)
C5	0.082 (5)	0.089 (5)	0.059 (4)	0.019 (4)	0.031 (4)	−0.005 (4)
C6	0.089 (5)	0.091 (5)	0.070 (5)	0.011 (4)	0.014 (4)	−0.033 (4)
C7	0.084 (5)	0.072 (5)	0.095 (6)	−0.016 (4)	0.021 (4)	−0.037 (4)
C8	0.069 (4)	0.061 (4)	0.068 (4)	−0.014 (3)	0.015 (3)	−0.015 (3)
C9	0.050 (3)	0.043 (3)	0.034 (2)	−0.008 (2)	0.010 (2)	0.0001 (19)
C10	0.076 (4)	0.048 (3)	0.049 (3)	0.004 (3)	0.013 (3)	−0.002 (2)
C11	0.124 (7)	0.041 (3)	0.059 (4)	−0.017 (4)	0.018 (4)	−0.005 (3)
C12	0.116 (7)	0.074 (5)	0.054 (4)	−0.049 (5)	0.026 (4)	−0.008 (3)
C13	0.070 (4)	0.103 (6)	0.059 (4)	−0.045 (4)	0.021 (3)	−0.011 (4)
C14	0.056 (3)	0.066 (4)	0.055 (3)	−0.012 (3)	0.021 (3)	−0.004 (3)
C15	0.034 (2)	0.049 (2)	0.052 (3)	0.005 (2)	0.0104 (18)	0.007 (3)
C16	0.043 (3)	0.067 (4)	0.076 (4)	−0.002 (2)	0.010 (3)	0.012 (3)
C17	0.046 (4)	0.094 (6)	0.130 (7)	0.007 (3)	0.038 (4)	0.029 (5)
C18	0.079 (5)	0.110 (6)	0.133 (8)	0.018 (5)	0.074 (5)	0.026 (7)
C19	0.128 (8)	0.101 (6)	0.074 (5)	0.036 (5)	0.047 (5)	0.001 (4)
C20	0.060 (4)	0.068 (4)	0.061 (4)	−0.002 (3)	0.023 (3)	−0.007 (3)
Sn2	0.03997 (16)	0.04791 (18)	0.04579 (17)	0.00040 (15)	0.00790 (13)	0.00084 (15)
Cl2	0.0441 (7)	0.0668 (8)	0.0983 (11)	0.0105 (8)	−0.0033 (7)	0.0098 (10)
S2	0.0523 (8)	0.0547 (7)	0.0489 (7)	0.0072 (6)	0.0076 (6)	−0.0020 (6)
O2	0.0425 (19)	0.056 (2)	0.059 (2)	0.0104 (17)	0.0079 (17)	−0.0024 (18)
C21	0.082 (5)	0.080 (5)	0.196 (11)	0.022 (4)	0.086 (7)	0.031 (6)
C22	0.081 (5)	0.070 (4)	0.070 (4)	0.020 (3)	0.020 (4)	0.015 (3)
C23	0.037 (2)	0.062 (3)	0.040 (2)	0.001 (2)	0.008 (2)	−0.002 (2)
C24	0.063 (4)	0.064 (3)	0.055 (3)	0.005 (3)	0.016 (3)	0.009 (3)
C25	0.085 (5)	0.087 (5)	0.073 (5)	−0.007 (4)	0.016 (4)	0.027 (4)
C26	0.062 (4)	0.163 (9)	0.050 (4)	−0.007 (5)	0.016 (3)	0.023 (5)
C27	0.071 (5)	0.139 (8)	0.050 (4)	−0.008 (5)	0.027 (3)	−0.019 (4)
C28	0.066 (4)	0.080 (5)	0.061 (4)	0.005 (3)	0.019 (3)	−0.011 (3)
C29	0.044 (3)	0.054 (3)	0.036 (2)	0.005 (2)	0.010 (2)	0.004 (2)
C30	0.060 (4)	0.078 (4)	0.055 (3)	0.002 (3)	0.023 (3)	0.012 (3)
C31	0.093 (5)	0.109 (6)	0.050 (4)	0.028 (5)	0.032 (4)	0.015 (4)
C32	0.104 (6)	0.088 (5)	0.050 (4)	0.026 (5)	0.005 (4)	−0.013 (4)
C33	0.089 (5)	0.076 (4)	0.057 (4)	−0.015 (4)	−0.004 (4)	−0.012 (3)
C34	0.062 (4)	0.070 (4)	0.051 (3)	−0.013 (3)	0.014 (3)	−0.007 (3)
C35	0.047 (3)	0.049 (3)	0.045 (3)	−0.003 (2)	0.007 (2)	−0.001 (2)
C36	0.059 (4)	0.060 (3)	0.057 (4)	0.003 (3)	0.007 (3)	−0.007 (3)
C37	0.080 (5)	0.049 (3)	0.063 (4)	0.000 (3)	0.003 (3)	−0.001 (3)
C38	0.084 (5)	0.059 (4)	0.078 (5)	−0.021 (4)	0.012 (4)	0.005 (3)
C39	0.065 (4)	0.066 (4)	0.137 (8)	−0.020 (3)	0.035 (5)	−0.003 (4)
C40	0.052 (4)	0.064 (4)	0.109 (6)	−0.001 (3)	0.026 (4)	−0.004 (4)

### Geometric parameters (Å, º)

**Table:**

Sn1—C15	2.115 (4)	Sn2—C29	2.127 (5)
Sn1—C9	2.118 (5)	Sn2—C23	2.130 (5)
Sn1—C3	2.125 (5)	Sn2—C35	2.147 (6)
Sn1—Cl1	2.4708 (14)	Sn2—O2	2.368 (4)
Sn1—O1	2.487 (4)	Sn2—Cl2	2.5061 (14)
S1—O1	1.505 (4)	S2—O2	1.510 (4)
S1—C1	1.697 (10)	S2—C22	1.757 (6)
S1—C2	1.758 (9)	S2—C21	1.784 (8)
C1—H1A	0.9600	C21—H21A	0.9600
C1—H1B	0.9600	C21—H21B	0.9600
C1—H1C	0.9600	C21—H21C	0.9600
C2—H2A	0.9600	C22—H22A	0.9600
C2—H2B	0.9600	C22—H22B	0.9600
C2—H2C	0.9600	C22—H22C	0.9600
C3—C4	1.381 (8)	C23—C28	1.378 (8)
C3—C8	1.383 (8)	C23—C24	1.386 (8)
C4—C5	1.379 (9)	C24—C25	1.388 (9)
C4—H4	0.9300	C24—H24	0.9300
C5—C6	1.373 (11)	C25—C26	1.351 (12)
C5—H5	0.9300	C25—H25	0.9300
C6—C7	1.362 (12)	C26—C27	1.364 (13)
C6—H6	0.9300	C26—H26	0.9300
C7—C8	1.384 (9)	C27—C28	1.385 (10)
C7—H7	0.9300	C27—H27	0.9300
C8—H8	0.9300	C28—H28	0.9300
C9—C10	1.381 (8)	C29—C34	1.377 (8)
C9—C14	1.390 (8)	C29—C30	1.377 (8)
C10—C11	1.405 (9)	C30—C31	1.375 (10)
C10—H10	0.9300	C30—H30	0.9300
C11—C12	1.364 (11)	C31—C32	1.386 (12)
C11—H11	0.9300	C31—H31	0.9300
C12—C13	1.359 (12)	C32—C33	1.370 (11)
C12—H12	0.9300	C32—H32	0.9300
C13—C14	1.391 (9)	C33—C34	1.385 (9)
C13—H13	0.9300	C33—H33	0.9300
C14—H14	0.9300	C34—H34	0.9300
C15—C16	1.381 (8)	C35—C36	1.366 (8)
C15—C20	1.387 (8)	C35—C40	1.391 (8)
C16—C17	1.375 (10)	C36—C37	1.381 (9)
C16—H16	0.9300	C36—H36	0.9300
C17—C18	1.348 (13)	C37—C38	1.351 (10)
C17—H17	0.9300	C37—H37	0.9300
C18—C19	1.386 (13)	C38—C39	1.361 (11)
C18—H18	0.9300	C38—H38	0.9300
C19—C20	1.382 (10)	C39—C40	1.386 (9)
C19—H19	0.9300	C39—H39	0.9300
C20—H20	0.9300	C40—H40	0.9300
			
C15—Sn1—C9	116.8 (2)	C29—Sn2—C23	114.4 (2)
C15—Sn1—C3	127.7 (2)	C29—Sn2—C35	119.8 (2)
C9—Sn1—C3	112.9 (2)	C23—Sn2—C35	124.7 (2)
C15—Sn1—Cl1	93.59 (13)	C29—Sn2—O2	86.73 (16)
C9—Sn1—Cl1	97.39 (15)	C23—Sn2—O2	86.23 (17)
C3—Sn1—Cl1	95.16 (14)	C35—Sn2—O2	86.50 (18)
C15—Sn1—O1	85.09 (16)	C29—Sn2—Cl2	93.96 (14)
C9—Sn1—O1	87.17 (17)	C23—Sn2—Cl2	92.53 (14)
C3—Sn1—O1	82.21 (17)	C35—Sn2—Cl2	94.06 (16)
Cl1—Sn1—O1	175.36 (11)	O2—Sn2—Cl2	178.75 (11)
O1—S1—C1	107.0 (5)	O2—S2—C22	105.6 (3)
O1—S1—C2	105.9 (4)	O2—S2—C21	104.8 (3)
C1—S1—C2	98.8 (5)	C22—S2—C21	98.2 (4)
S1—O1—Sn1	120.6 (2)	S2—O2—Sn2	133.4 (2)
S1—C1—H1A	109.5	S2—C21—H21A	109.5
S1—C1—H1B	109.5	S2—C21—H21B	109.5
H1A—C1—H1B	109.5	H21A—C21—H21B	109.5
S1—C1—H1C	109.5	S2—C21—H21C	109.5
H1A—C1—H1C	109.5	H21A—C21—H21C	109.5
H1B—C1—H1C	109.5	H21B—C21—H21C	109.5
S1—C2—H2A	109.5	S2—C22—H22A	109.5
S1—C2—H2B	109.5	S2—C22—H22B	109.5
H2A—C2—H2B	109.5	H22A—C22—H22B	109.5
S1—C2—H2C	109.5	S2—C22—H22C	109.5
H2A—C2—H2C	109.5	H22A—C22—H22C	109.5
H2B—C2—H2C	109.5	H22B—C22—H22C	109.5
C4—C3—C8	118.0 (5)	C28—C23—C24	118.5 (6)
C4—C3—Sn1	118.1 (4)	C28—C23—Sn2	121.6 (5)
C8—C3—Sn1	123.9 (4)	C24—C23—Sn2	119.8 (4)
C5—C4—C3	121.8 (6)	C23—C24—C25	120.3 (7)
C5—C4—H4	119.1	C23—C24—H24	119.9
C3—C4—H4	119.1	C25—C24—H24	119.9
C6—C5—C4	118.7 (7)	C26—C25—C24	120.4 (8)
C6—C5—H5	120.7	C26—C25—H25	119.8
C4—C5—H5	120.7	C24—C25—H25	119.8
C7—C6—C5	121.0 (7)	C25—C26—C27	120.1 (7)
C7—C6—H6	119.5	C25—C26—H26	120.0
C5—C6—H6	119.5	C27—C26—H26	120.0
C6—C7—C8	119.7 (7)	C26—C27—C28	120.4 (7)
C6—C7—H7	120.1	C26—C27—H27	119.8
C8—C7—H7	120.1	C28—C27—H27	119.8
C3—C8—C7	120.7 (7)	C23—C28—C27	120.3 (7)
C3—C8—H8	119.6	C23—C28—H28	119.8
C7—C8—H8	119.6	C27—C28—H28	119.8
C10—C9—C14	118.8 (5)	C34—C29—C30	117.7 (5)
C10—C9—Sn1	120.9 (4)	C34—C29—Sn2	120.3 (4)
C14—C9—Sn1	120.3 (4)	C30—C29—Sn2	122.0 (5)
C9—C10—C11	119.7 (7)	C31—C30—C29	120.9 (7)
C9—C10—H10	120.2	C31—C30—H30	119.5
C11—C10—H10	120.2	C29—C30—H30	119.5
C12—C11—C10	121.1 (7)	C30—C31—C32	120.6 (7)
C12—C11—H11	119.5	C30—C31—H31	119.7
C10—C11—H11	119.5	C32—C31—H31	119.7
C13—C12—C11	119.2 (6)	C33—C32—C31	119.3 (7)
C13—C12—H12	120.4	C33—C32—H32	120.4
C11—C12—H12	120.4	C31—C32—H32	120.4
C12—C13—C14	121.2 (7)	C32—C33—C34	119.2 (7)
C12—C13—H13	119.4	C32—C33—H33	120.4
C14—C13—H13	119.4	C34—C33—H33	120.4
C9—C14—C13	120.1 (6)	C29—C34—C33	122.2 (6)
C9—C14—H14	120.0	C29—C34—H34	118.9
C13—C14—H14	120.0	C33—C34—H34	118.9
C16—C15—C20	118.6 (5)	C36—C35—C40	117.3 (6)
C16—C15—Sn1	123.7 (4)	C36—C35—Sn2	121.4 (4)
C20—C15—Sn1	117.7 (4)	C40—C35—Sn2	121.3 (5)
C17—C16—C15	120.8 (7)	C35—C36—C37	122.2 (6)
C17—C16—H16	119.6	C35—C36—H36	118.9
C15—C16—H16	119.6	C37—C36—H36	118.9
C18—C17—C16	120.5 (7)	C38—C37—C36	119.2 (7)
C18—C17—H17	119.8	C38—C37—H37	120.4
C16—C17—H17	119.8	C36—C37—H37	120.4
C17—C18—C19	120.3 (7)	C37—C38—C39	121.2 (7)
C17—C18—H18	119.8	C37—C38—H38	119.4
C19—C18—H18	119.8	C39—C38—H38	119.4
C20—C19—C18	119.6 (8)	C38—C39—C40	119.3 (7)
C20—C19—H19	120.2	C38—C39—H39	120.3
C18—C19—H19	120.2	C40—C39—H39	120.3
C19—C20—C15	120.2 (7)	C39—C40—C35	120.8 (7)
C19—C20—H20	119.9	C39—C40—H40	119.6
C15—C20—H20	119.9	C35—C40—H40	119.6

### Hydrogen-bond geometry (Å, º)

**Table:**

*D*—H···*A*	*D*—H	H···*A*	*D*···*A*	*D*—H···*A*
C1—H1*B*···Cl2^i^	0.96	2.83	3.555 (9)	134
C21—H21*B*···Cl1^ii^	0.96	2.82	3.716 (9)	156
C22—H22*B*···Cl1^ii^	0.96	2.94	3.813 (7)	153

Symmetry codes: (i) *x*+1, *y*, *z*+1; (ii) −*x*+1, *y*−1/2, −*z*+1.
